# Could Lithium Be Preserved for the Stabilization of Bipolar Patients?

**DOI:** 10.3390/ph19040527

**Published:** 2026-03-25

**Authors:** Paul Grof

**Affiliations:** Department of Psychiatry, Toronto University, Toronto, ON M5T 1R8, Canada; paulgrof75@gmail.com

**Keywords:** bipolar disorders, lithium, side-effects, monitoring

## Abstract

Lithium remains endorsed as first-line treatment for bipolar disorders across major clinical guidelines, yet robust evidence demonstrates its progressive decline in use in psychiatric practice across numerous countries. To justify this decline, concerns regarding lithium’s efficacy, safety profile, and monitoring requirements are frequently cited. Yet these apprehensions largely stem from an insufficient understanding of lithium’s clinical uses. In fact, when patients are selected for lithium stabilization according to a characteristic clinical profile and not just a bipolar verdict, lithium continues demonstrating good efficacy compared to all other psychiatric medications currently available. Moreover, after sufficient clinician and patient education regarding lithium stabilization principles, monitoring requirements stop being burdensome. Furthermore, among lithium-responsive patients, adverse effects are typically mild and clinically manageable, except for glomerular filtration rate decline, which tends to develop after decades of continuous administration. Thus, it might be possible to reverse this unfortunate decline in lithium’s use by teaching clinicians to identify the patient profile responsive to lithium stabilization and by implementing educational programs regarding optimal lithium utilization for psychiatrists, patients, and their families. Furthermore, it is worth investigating intermittent lithium administration to mitigate renal complications.

## 1. The Declining Use of Lithium

There was then and still is now no theoretical basis for lithium’s use, no rationale that could then or can now be used to sell it. As a result, the use of lithium will almost certainly end when Schou dies. Another agent, probably of lesser efficacy, will displace it by virtue of a marketing strategy that depends on offering a “biological rationale”…

David Healy, MD, The Creation of Psychopharmacology, 2002.

Fortunately, David Healy’s prophesy was not correct, but lithium’s use has certainly been moving in this direction. What can we, clinicians and researchers, do to reverse this trend?

Since the 1960s, lithium treatment has protected countless lives of patients suffering from bipolar disorders [1] and normalized psychosocial functioning, and has generated substantial healthcare savings [2]. However, recent decades have witnessed a paradoxical situation: while clinical guidelines consistently describe lithium as first-line treatment for bipolar disorders, its utilization has progressively declined across multiple countries [3,4]. The evidence of this decline is robust and highlights significant obstacles to continued adoption [5,6]. There may be, however, other factors indirectly influencing the decreasing therapeutic use of lithium. These factors include increasing lithium’s industrial use [7].

The reduction in lithium use has been particularly dramatic in the United States and pronounced throughout Western countries. However, lithium continues to be utilized in developing nations, presumably because it is inexpensive. For instance, recent data from the United States demonstrate that lithium use among patients with bipolar disorder decreased significantly from greater than 30% to below 15% over the past three decades. Although the European decline is less pronounced [3], both regions exhibit similar downward prescribing trends.

The main factors underlying this trend include concerns regarding lithium’s adverse effects [8], unjustified but lingering doubts about its efficacy [9], the need for closer monitoring [10], and perceived management difficulties. Additional factors include aggressive marketing of second-generation antipsychotics, which demonstrate comparable efficacy in treating acute manic episodes, and limitations in lithium’s commercial appeal [11]. Clinician preferences and attitudes toward lithium have been further influenced by patient and professional misinformation and beliefs [5].

These factors combined contribute to the decreasing trend in lithium utilization for treating bipolar disorder and related conditions. While lithium use has experienced temporary fluctuations previously following misguided publications between 1978 [12] and 2000 [13]—the current trend appears more sustained and ominous. If this trajectory continues without successful intervention, millions of patients with bipolar disorder and their families will suffer unnecessarily, with thousands never achieving stability and potentially losing their lives.

## 2. The Decline Likely to Worsen

Without intending to sound alarmist, there are justified reasons to anticipate that this decline in lithium use may accelerate. During the 1970s and 1980s, thousands of patients enthusiastically initiated lithium maintenance therapy. But after decades of continuous treatment, many of these patients begun exhibiting adverse renal effects [14].

Consequently, clinicians became increasingly aware of these complications, recognized the renal adversity in greater numbers of patients, and expressed concern regarding patient welfare, as well as potential medicolegal challenges. These developments have the potential to speed up lithium’s disappearance from clinical practice, particularly in Western countries.

## 3. Misunderstood Efficacy

Over the years, the efficacy of lithium stabilization has been repeatedly questioned because of a misunderstanding. The underlying confusion stems from the radical shift in diagnosing bipolar disorders after the discovery of lithium’s stabilizing value. Incontrovertible evidence of lithium’s efficacy derived from double-blind trials was conducted by investigators who were trained to diagnose according to the Kraepelinian tradition [15]. Those clinicians identified manic-depressive illness according to the patient’s clinical profile, with particular attention to the fully episodic course and supportive findings in family history.

But later, the diagnostic fashion changed. Diagnosing bipolar disorder exclusively according to the DSM symptom-based criteria [16] (Diagnostic and Statistical Manual of Mental Disorders) gradually prevailed and very significantly expanded diagnostic boundaries. It is relevant to explain briefly how this shift unfolded and changed lithium’s use [17,18].

When Schou and Baastrup first reported lithium’s remarkable stabilizing effects [19,20], no other medication could influence manic-depressive recurrences. Once lithium stabilization was approved by regulatory agencies, suddenly, regained mood stability was observed in thousands of manic-depressive patients who had previously suffered for decades, spending considerable time hospitalized. With lithium maintenance, these patients suddenly returned to their families and work, maintaining long-term stability—an outcome that then seemed miraculous.

Naturally, this extraordinary observation generated much hope, as well as extensive experimentation, in patients with difficult-to-treat psychiatric disorders. With much optimism, many such illnesses were tried on lithium. Lithium was subsequently evaluated in patient cohorts with 15 different diagnoses [21]. Most did not respond to lithium. However, complete stabilization was achieved in classical manic-depressive illness, with some benefits detected also in schizoaffective and schizophreniform disorders [22,23,24] and cycloid psychoses [25]—three conditions sharing mood abnormalities in their clinical presentation. The conclusion drawn was that these three conditions represent misdiagnosed bipolar disorders, with any lithium benefit wrongly equated to bipolar disorder.

In parallel with the change in diagnostic fashion, lithium’s apparent efficacy gradually diminished and eventually seemingly vanished [13,17]. This oversimplified diagnostic reasoning (diagnosis by DSM symptoms as opposed to the patient’s clinical profile) unfortunately overlooked that lithium offers psychiatric patients a variety of ten benefits that are qualitatively different from the stability achieved in the classical type of manic-depressive patients [26]. For example, in the episodically recurring bipolar type, lithium prevents both manias and depressions successfully; discontinuation produces no rebound phenomenon [27,28], the lithium response is fully reproducible after restarting lithium, and the anti-suicide effect is well documented [29,30,31].

Patients with other bipolar spectrum subtypes benefit differently: lithium only helps with manias but not depressions, discontinuation often precipitates rapid and exaggerated rebound, and the gain from lithium becomes poorly reproducible after discontinuation [32]. The benefits more closely resemble those of neuroleptics.

These qualitative differences were unfortunately ignored, and the diagnostic fashion reformed to the detriment of lithium’s reputation as a highly effective stabilizer. As bipolar diagnosis evolved from a Kraepelinian clinical profile to DSM III to V symptom criteria [33], the incidence of bipolar diagnoses markedly increased, while lithium’s efficacy became increasingly questioned [17,18]. By 2000, maintenance clinical trials emerged that included these loosely diagnosed bipolar disorders and found lithium not only ineffective but somewhat worse than placebo [13].

## 4. Reassessing Monitoring Requirements

Like many pharmacological substances, lithium can be toxic if used inappropriately. Therefore, out of caution, during the 1960s and 1970s, frequent determinations of lithium plasma concentration were required [34]. However, these guidelines were not sufficiently adjusted as clinical practice evolved.

Over time, Mogens Schou concluded that for long-term treatment, the critically important safety factor is sufficient patient education about the principles of lithium stabilization and compliance. He therefore developed and updated educational materials [35,36], explaining essential knowledge for patients and relatives regarding safe and effective lithium treatment.

Through six decades of treating with lithium, we have found that with proper medical assessment, correctly adjusted individual dosing, and sufficient patient education, blood value monitoring, including lithium levels every 6–12 months or when clinically indicated, is sufficient. This observation was made in lithium-responsive patients.

Lithium’s potential toxicity is often misconstrued. The main risk emerges when lithium accumulates in tissues following prolonged intake or interacts with medical co-medication. Otherwise, in the correct dosage provided to patients who are sufficiently medically screened, lithium is generally well tolerated.

Critical elements for safe lithium treatment include careful medical patient selection, correct determination of individual patient dosage, sufficient knowledge of pharmacological data, and patient education about the principles of lithium stabilization and medication compliance [37].

## 5. Rethinking Side Effects

Misunderstandings also exist regarding lithium treatment adverse effects. Lithium’s adverse effects depend on numerous administration factors: formulation type (plain versus slow-release) [38], dosing amount and frequency [39], patient factors such as age and bipolar subtype, and variables known from general pharmacology.

Attention also needs to be paid to side effect differences between lithium stabilization responders and non-responders. Significant differences exist in adverse effects between these two groups. In a cohort of three hundred lithium maintenance responders, the most frequent complaint was increased thirst in one-third of patients, primarily at treatment onset [40]. Other lithium side effects were mild, often transitory, present in only 10% of patients, and often correctable. The only troublesome, disconcerting side effect was reduced glomerular filtration, becoming significant after decades of uninterrupted administration [8,14].

The above observation differs markedly from the adverse effects reported in undifferentiated lithium-treated populations [8], which fail to consider the above-mentioned factors: e.g., whether dosage was appropriate, whether lithium levels were therapeutic, whether lithium was properly indicated, or whether patients responded.

Virtually all observations have been collected and reported indiscriminately, combining all these different situations together. Elevated lithium side effects in such reports justifiably raise concerns [41], but they are not informative for properly run clinical practice. Included in these reports are the lithium side effects of patients who do not respond and should not continue with lithium maintenance, which lacks clinical utility. In my opinion, the particularly clinically informative has been published by Tondo et al. [42].

## 6. How to Reverse the Trend

Introducing long-term lithium treatment into clinical practice was challenging [1]. It is therefore worrisome to see the declining use of lithium in bipolar disorders, as it represents a significant loss for both patients and clinical practice. Yet this troubling development may be reversed through strategic interventions that would address the core obstacles that mistakenly limit lithium’s utilization in practice. Such a path forward would require an approach encompassing improved patient selection for maintenance, innovative kidney protection strategies, and enhanced education across patients, families, and clinicians.

## 7. Selecting Lithium Responders in Advance

The first step in reversing this trend would rest in helping clinicians appreciate that a positive lithium maintenance response can be predicted with remarkable accuracy from each patient’s clinical profile [43,44]. The ability to forecast lithium stabilization success emerged from five decades of systematic research, supported in particular by discriminant function [44] and machine learning (AI) analyses [31]. Utilizing this recognition markedly improves lithium treatment outcomes, reduces side effects, and simplifies clinical practice. The International Group for the Study of Lithium-Treated Patients (IGSLIs) continues to advance in this direction, currently designing an extensive collaborative study to incorporate established genetic correlates of response alongside clinical predictive features. Before identifying lithium maintenance responders in advance in clinical practice, carefully designed prospective studies are needed.

Successful predictions from the patient’s clinical profile depend primarily on the accurate evaluation of two factors: the type of clinical course and family history findings. However, these two predictors need to be defined in a distinctive way that is often overlooked in current clinical practice [44,45].

The most important predictor from the clinical course is that true “illness-free intervals” must be differentiated from “clinical recovery.” An episodic bipolar course with psychopathology-free intervals requires a return to ordinary mental health. This differs substantially from “clinical recovery,” where patients return to family and work responsibilities, often without complaining of any symptoms, yet closer clinical assessment reveals persistent fluctuations in anxiety, mood, sleep, cognition, and other residual symptoms. Only true free intervals predict a lasting lithium-stabilizing response.

To be predictive, family history assessment extends beyond information patients provide and needs to include reports from other family members, particularly a relative who possesses more comprehensive knowledge about psychiatric problems and mental health in the family. The identification of psychiatric disorders with episodic courses contributes significantly to an accurate prediction of lithium stabilization [46,47].

When the above assessment principles are applied, approximately one-third of patients who are currently broadly diagnosed as bipolar can be identified as having a lithium stabilization-responsive profile [48]. Some patients have bipolar propensity and lithium responsiveness, yet for a long time may present only with recurrent depression or recurrent disabling anxiety. The clinical implications of improved patient selection for lithium stabilization extend far beyond treating an individual patient. Once clinicians witness how identifying stabilization response in advance simplifies further clinical care and transforms patients’ lives, their appreciation of lithium stabilization is likely to improve and may start reversing the downward trend in lithium use.

Observing the lasting stability in patients appropriately selected for lithium stabilization motivates clinicians to abandon the prevailing trial-and-error approach to mood stabilization and reduce excessive, unjustified polypharmacy that leads to frustrating outcomes and higher side effects. Treating bipolar patients who are lithium-stabilized and remain that way [49] liberates clinicians’ time and resources for attending to the remaining two-thirds of bipolar spectrum patients who require different treatment strategies. Such experiences with lithium-stabilized patients also offer a clinical hint that lithium is usually a poor choice for successful maintenance treatment of other types of bipolar spectrum disorders.

It may be of interest that the patient’s clinical profile, which was found to be linked with satisfactory stabilization response, tends to overlap with the description that Kraepelin provided for manic-depressive illness [15]. Also, the clinicians who, during the 1970s, completed successful double-blind lithium studies were diagnosing within the Kraepelinian tradition. The meta-analyses that now demonstrate the efficacy of lithium stabilization always carefully involve such studies [50], and they probably would not demonstrate lithium’s efficacy without these studies.

## 8. Protecting Patients’ Kidney Functions

Parallel to improving patient selection, protecting the patient’s kidney function would represent another crucial step in preserving lithium treatment for bipolar patients. Schou et al. recommended continued daily lithium maintenance [51], as they were aware that the manic-depressive course is recurrent, that recurrences reappear capriciously, and that the cycle length is highly variable in individual patients. They advised lithium treatment without any interruption because they worked mostly with severely ill patients who were finally stabilized on lithium for the first time in their lives, and the negative effect on kidney function was not known.

There was not much reason to worry then. In fact, after the first decade of lithium stabilization, an abnormally low glomerular filtration rate (referred to as GFR) was observed only in those individuals who showed abnormally low GFR during the medical screening for lithium treatment [52]. The GFR values started dropping substantially after some decades of continuous lithium intake.

Professor Rybakowski’s team provided an important example through their unique report of five patients treated with lithium for over fifty years [14]. Despite receiving exemplary conservative lithium treatment with careful monitoring, patients developed GFR disruptions. I witnessed the quality of their care when I was allowed to review this bipolar cohort during my visit on behalf of an IGSLI collaborative study. Rybakowski’s team’s findings demonstrated again that lithium, regardless of expert management quality, possesses inherent nephrotoxic potential that manifests after decades of uninterrupted use.

We had similar observations in our Ontario bipolar patient cohorts. After the first decades of continuous lithium treatment, our lithium stabilization responders showed no demonstrable adverse effects on GFR [52] and only mildly increased urinary volume. The GFR decrease became evident after three decades of uninterrupted lithium treatment and exacerbated after four and five decades. Over one-fifth of our patients stabilized with lithium for more than four decades exhibited abnormally low GFR and, according to our nephrologists, attributable solely to lithium treatment [53].

However, learning more about the episodic course of classical manic-depressive illness opened new possibilities for kidney protection through intermittent treatment strategies. In the 1960s, with Professor Jules Angst, we gathered data from lifelong clinical courses of manic-depressive patients. At that time, it was still possible to obtain records from a large cohort of patients who were medicated only during the acute episodes and received no maintenance treatment [54]. Later, clinical course observations were added from bipolar patients free of mood stabilizers during their initial episodes.

For two decades, we then explored what may drive this seemingly capricious, recurrent process of bipolar illness. Finally, led by neuroscientists Anna Yamamoto and Josef Lat [55,56,57,58], we uncovered during the clinical courses largely predictable oscillatory patterns that neuroscientists had described in extensive explorations in animal brains.

The findings indicate that bipolar recurrences reflect brain oscillatory processes with excessive amplitudes poorly modulated. Clinicians easily miss this because observing natural, untreated courses of bipolar illness has now become a rarity. The brain is a huge assembly of oscillators active on all different neuronal levels. Because of the quality of neurons in the frontocentral areas [59], oscillations quickly expand and retract, and the activity above the usual ceiling may take place in episodes. In general, these oscillatory patterns are either linear or exponential, but otherwise individual.

The oscillatory nature of bipolar illness can be demonstrated retrospectively through sufficient clinical information or prospectively using serial dexamethasone suppression tests as brain arousal level proxies. This understanding enables the identification of individual high-risk and low-risk periods, creating opportunities for treatment interruptions during suitable periods. Intermittent treatment strategy retains lithium stabilization while it provides protection by reducing cumulative kidney exposure to lithium. Using this strategy, it is important not to underestimate the risk of recurrence reflected in the literature [60,61,62].

We observed that in patients with severe illness, recurrences develop during the peak of each cycle. In these patients, we first noticed the relationship between the oscillatory processes and recurrences. In patients with less severe classical bipolar illness, only the majority of the cycle peaks are associated with recurrences (presumably because of other biological and psychosocial factors at the time). However, the subsequent recurrences and neurobiological measurements indicated that in these less severe patients, their individual cycle lengths continue unchanged. In other words, the individual cycle pattern for each patient remained unchanged. Therefore, high- and low-risk time periods may be estimated and, in selected lithium responders, make intermittent treatment possible.

This lack of adaptability appears to be one of the characteristics of classical bipolar course; unless it is artificially sped up, e.g., by antidepressants. In comparison, in healthy subjects, the brain oscillatory patterns are adaptive, variable, and change under the circumstances [63]. More details of this approach are explained with examples in other publications [64] and in an upcoming book.

Successful and beneficial use of intermittent treatment for specific situations is not new in medicine and has been applied in chronic pain management, cardiology and psychiatry [65,66]. Our clinical experience with intermittent lithium treatment spans thirty-five years and has included thirty-one carefully selected patients during long-term lithium treatment [53].

Patients requesting intermittent lithium treatment had to meet a set of criteria to be considered suitable: a clearly documented fully episodic course; good response to continuous lithium maintenance for a sufficient time; the patient’s significant other cooperating and consenting to the treatment plan; and feasible regular clinical supervision. These lithium-responsive patients typically requested a modification of their continuous lithium treatment to intermittent after they learned about lithium’s potential nephrotoxicity. Therefore, these patients embody an extended clinical observation, not a representative sample. I need to stress the point mentioned above: these patients were all excellent responders to lithium maintenance.

The patients were placed on intermittent lithium treatment for clinical purposes, and no comparative study was then intended. Yet the preliminary findings in this pilot are promising for kidney protection. To obtain some quantitative indication, the age correlations of patients on intermittent treatment were compared with those of patients on continuous lithium and patients who never received lithium.

Preliminary analyses imply that in patients treated intermittently, GFR decrease with age resembles that of lithium-free controls. Thus, intermittent lithium treatment may avoid the excessive effects of uninterrupted, continuous long-term lithium treatment. The correlation between GFR decline and age in intermittent treatment patients (r = 0.37, N = 31) closely matches lithium-free controls (r = 0.39, N = 100), while continuous treatment patients indicate much higher GFR decline (r = 0.68, N = 110).

There is a widespread belief that discontinuing lithium medication is a high-risk step, often followed by a quick relapse, even with an intensity worse than ever before. This is true for bipolar spectrum disorders but does not apply to the classical type of bipolar illness. Here, the relapse comes only as one would expect from the previous course of the illness [27,28].

Over the decades of working with intermittent lithium treatment, bipolar depressive recurrences were observed in only three patients, all of them occurring when the patient stayed off lithium longer than they were advised, according to the duration of the low-risk period. Fortunately, in lithium stabilization responders, the lithium benefit can be promptly reproduced following discontinuation. After resuming their lithium dosage, all three patients fully recovered within 10–14 day [67].

These observations, while preliminary and obtained in specialized clinical settings, suggest that lithium’s GFR effects might result from uninterrupted long-term exposure rather than from lithium itself. To test this possibility, other factors such as, for example, cumulative lithium dose, need to be included in a future study. The pilot data presented here need to be considered hypothesis generating. For episodic bipolar illness, intermittent treatment offers hope for maintaining therapeutic benefits while avoiding the adverse effects of decades-long continuous exposure.

Alternative strategies for assessing recurrence risk are also being developed [68,69]. Furthermore, lowering the lithium dosage during low-risk periods rather than fully discontinuing also merits further exploration. If effective, some clinicians may prefer such less radical approaches to kidney protection. Moreover, in bipolar patients exhibiting inflammatory markers [70,71], it is important to consider adding an anti-inflammatory medication and reducing the lithium dosage to help protect the kidneys.

## 9. Improved Education

Finally, to reverse the trend, much needs to be done to educate psychiatrists, their patients, and family members about the principles of lithium stabilization and suicide prevention. It would be helpful to correct the prevailing beliefs and misconceptions about efficacy, side effects, and adequate monitoring of lithium stabilization.

## 10. Conclusions

Bipolar disorders afflict millions of individuals and are potentially lethal. Reversing lithium’s declining clinical use through the outlined strategies would serve the broader goal of improving bipolar disorder treatment and reducing the great burden of these devastating illnesses on patients and their families.

When lithium is used appropriately in carefully selected patients with adequate protection and monitoring, it offers unique, unparalleled mood stability, suicide protection, and quality of life improvement. Restoring this treatment option through strategic interventions may represent a critical step toward optimizing care for all patients across the bipolar spectrum.

## Data Availability

The clinical data are located in the Medical record Departments of Hamilton Psychiatric Hospital, Hamilton, and Royal Ottawa Hospital, Ottawa, ON, Canada.
